# Peer Review of “Are We Sure We Fully Understand What an Infodemic Is? A Global Perspective on Infodemiological Problems”

**DOI:** 10.2196/40822

**Published:** 2022-07-21

**Authors:** Gunther Eysenbach

**Keywords:** communication, conspiracy, COVID-19, education, fake news, infodemic, infodemiology, mass media, public health, risk perception, science

*This is a peer-review report submitted for the paper “Are We Sure We Fully Understand What an Infodemic Is? A Global Perspective on Infodemiological Problems.”*

## Round 1 Review

### General Comments

This is a reasonable viewpoint/opinion paper [1]. I do not agree with everything that is being said but that is also not the goal—it is the authors’ opinion.

I do think the paper should be transferred to JMIR Infodemiology. As to the authors’ statement that “the obsessive pursuit of prestige must be drastically limited as they undermine the credibility of science,” I agree, and that also extends to obsession with the impact factor, so I hope the author follows his own advice and agrees to a transfer.

### Specific Comments

#### Major Comments

1. It may be worth citing [2] in addition to ref 1.
2. Preprint servers do screen submissions, and there are different levels of screening, varying by preprint server. For example, MedRxiv implemented more strict criteria on COVID-19 compared with Zenodo, etc.
3. “Level of evidence” is a well-known phrase and is typically thought of in terms of study type rather than dissemination modality (ie, “systematic review” is better than “RCT,” which is better than “observational studies,” etc). If you come up with a new hierarchy—that is not directly speaking to the study type—I would suggest you come up with a new phrase or label for the type of hierarchy you are suggesting.
